# Rapid Determination of Diclofenac in Pharmaceutical Formulations by Capillary Zone Electrophoresis

**DOI:** 10.3797/scipharm.1201-02

**Published:** 2012-02-23

**Authors:** Bodo Lachmann, Martin Kratzel, Christian R. Noe

**Affiliations:** Department of Medicinal Chemistry, University of Vienna, Althanstraße 14, 1090 Vienna, Austria

**Keywords:** Capillary Zone Electrophoresis, CZE, Diclofenac Sodium, Pharmaceutical Formulations, CE

## Abstract

Capillary electrophoresis is competitive to HPLC and other chromatographic methods, predominantly when charged analytes have to be separated. The time of analysis can be reduced by the use of very short capillaries applying a high voltage. In most instruments which are commercially available the so-called ‘short end’ of the capillary can be used for separation, leading to very rapid separations. In this contribution we want to demonstrate this approach by using Diclofenac Sodium as an analyte.

## Introduction

**Fig. 1.**
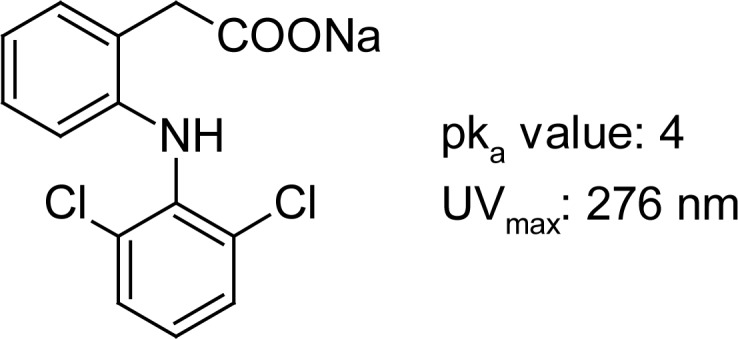
Diclofenac Sodium

Diclofenac Sodium (Fig. 1) is one of the most popular nonsteroidal anti-inflammatory drugs, which is commonly used in different dosage forms, such as tablets, ointments or injections. It acts as a potent inhibitor of prostaglandin synthesis [1] with pronounced antiinflammatory, analgesic and antipyretic properties. Several methods for the determination of this drug in pharmaceutical preparations or biofluids have been reported in the literature. The most favoured ones involve HPLC [2, 3] or spectrophotometric and colorimetric [4] methods. During the last two decades, capillary electrophoresis has become a very suitable analytical tool for a wide range of compounds [5]. Capillary electrophoresis has become especially popular in the field of pharmaceutical analysis [6]. Different methods have been published for the determination of diclofenac by capillary electrophoresis in body fluids [7] and in pharmaceutical preparations [8]. For the determination in human urine a method with electrochemical detection was established, using a phosphate buffer as background electrolyte. Donato et al. used for the quantification in different pharmaceuticals either micellar electrokinetic capillary chromatography or capillary zone electrophoresis with UV-detection.

The aim of our work was on the one hand to develop a short and simple CZE-method which allows the determination of diclofenac in several dosage forms, such as tablets, ointments or injections. On the other hand the use of very short capillaries should be evaluated in commercially available instruments, e. g. injection from the outlet sample tray and separation by reversed polarity. For both methods an alkaline borate buffer system containing 25 mM sodium tetraborate, pH 9.3 and UV detection at either 200 nm or 214 nm was used.

## Results and Discussion

Starting with a tetraborate buffer of 50 mM, method development has taken place by modifying several parameters in the ‘normal’ polarity mode. In order to avoid high current values, the concentration of the buffer system has been reduced. The 25 mM buffer shows the best performance, lower concentrated solutions resulted in less reproducible migration times and reduced time of use. Changing wavelength from 200 nm to 214 nm resulted in no drastical loss of sensitivity, but a significantly smoother baseline has been obtained. In order to shorten the time of analysis, the several rinsing steps have been also optimized. The approach to rinse only with running buffer between the runs has not affected reproducibility of migration times, as long as an additional rinsing step has been performed every 20 runs. Applying the same parameters in the ‘reversed’ polarity mode, a separation in less than 1 min becomes feasible. To receive the same amount of data points as in method 1, acquisition rate has been doubled. A crucial aspect of this ‘reversed’ method is the rinsing direction: rinsing in ‘normal’ direction causes less reproducible peak areas for the ‘reversed’ injection from the outlet sample tray.

For both methods, nearly similar calibration curves have been obtained (y = 2.3896x + 0.0726, R2 = 0.9983, method 1), (y = 2.2955x + 0.0654, R2 = 0.9986, method 2). Standard deviations of the migration times and the corrected peak areas were in both cases less than 1.5% (n=10). To check intraday and interday precision, one of the sample solutions has been analyzed on three consecutive days with both methods. No statistically significant deviation has been obtained. The sample solutions have been shown to be stable for over 72 hours.

The results obtained by method 2 for several dosage forms are shown in table 1. Only in the case of the Voltaren Emulgel^®^, the approach of the direct analysis of the aqueous solution was not possible; in this case, a further purification step of the sample solution seems to be necessary. We plan to prove in the future whether the methods presented here can also be applicable for the determination of potential degradation products or side products of the synthesis. The use of benzoic acid as internal standard heightens roughness of the method and prevents possible deviations caused by an unstable injection system.

In this contribution, we present a very rapid and simple method for the determination of diclofenac using an alkaline borate buffer system containing 25 mM sodium tetraborate, pH 9.3. With an effective capillary length of 10 cm and an applied voltage of 28 kV, separation can be performed in less than 1 min. Quantification was achieved by using benzoic acid as an internal standard. Calibrations curves were linear between 0.1 and 1.5 mg/ml with RSD values between 0.5 and 3.6%. Recovery rates of 99.2% were obtained in average. For the determination in pharmaceutical preparations, no sample cleanup was necessary, the samples were solved in water, sonicated, and aliquots of this solution were further diluted. Both methods (effective capillary length of 10 cm vs. 20 cm) have been proven to be satisfactory, no significant differences have been found.

## Experimental

### Equipment

All separations were performed using a P/ACE MDQ capillary electrophoresis system with an UV detector (Beckman Instruments, Munich). Photometric on-column detection was carried out at 214 nm, with a data rate of 4 Hz in case of normal polarity and 8 Hz in case of reversed polarity. ‘32 Karat^®^’ software (Beckman Instruments, Munich) was applied for data acquisition and analysis.

### Electrophoretic conditions

Uncoated fused-silica capillaries (Beckman eCap) of 50 μm I. D. (385 μm O. D.) were used for all separations. A capillary of a total length of 30 cm was used for the separations. The detector was situated either 10 cm or 20 cm from the cathodic end (normal polarity). Every 20 runs the capillary was flushed 1 min with 1 N NaOH and 1 min with 2 N HCl, both 50 psi, separation was carried out at 20 °C (Beckman capillary cartridge coolant).

### Time programs

#### Method 1

- Rinsing the capillary with the BGE for 30 sec (30 psi)
- Injection of the sample for 3 sec (0.5 psi)
- Applying +28 kV for 2 min – Cartridge temperature 20 °C
- Monitoring at 214 nm with a data rate of 4.0 Hz

#### Method 2

- Reverse rinsing with BGE 30 sec (30 psi) reverse
- Reverse injection of the sample for 3 sec (0.5 psi)
- Applying −28 kV for 1 min – Cartridge temperature 20 °C
- Monitoring at 214 nm with a data rate of 8.0 Hz

### Chemicals and Buffers

The buffer used for electrophoretic separation was prepared by dissolving sodium tetraborate in HPLC-grade water and adjusting the pH to 9.3 with 10 M NaOH or 10 M HCl. Diclofenac Sodium standard was supplied by Fährhaus Pharma, Germany, the several dosage forms were a friendly gift from Novartis Austria.

### Sample and standard preparation

#### Standard solutions

Accurately weighed diclofenac was transferred into a 100 ml flask, and 20.0 ml of a standard solution of sodium benzoate (1.6205 g / 500 ml water) were added. The flask containing this solution was filled to the appropriate volume with a mixture of 9 parts water and 1 part BGE. Aliquots of this solution were diluted 1:5 with water prior to injection.

#### Sample solutions

Tablets: One tablet was weighed and afterward transferred into a 100 ml flask. 20.0 ml of a standard solution of sodium benzoate (1.6205 g / 500 ml water) were added. The flask containing this solution was filled to the appropriate volume with a mixture of 9 parts water and 1 part BGE.

Other preparations: An aliquot of the diclofenac containing gel or solution was weighed and transferred in a 100 ml flask and then 20.0 ml of a standard solution of sodium benzoate (1.6205 g / 500 ml water) were added. The flask containing this solution was filled to the appropriate volume with a mixture of 9 parts water and 1 part BGE.

## Authors’ Statement

### Competing Interests

The authors declare no conflict of interest.

## Figures and Tables

**Fig. 2.**
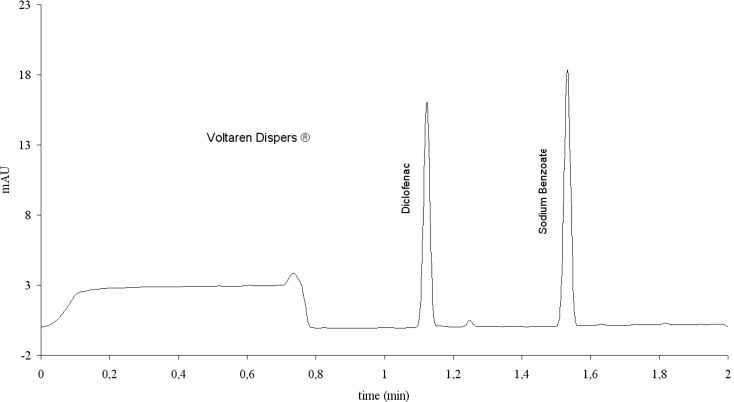
Electropherogram of a Voltaren Dispers® sample, obtained with method 1.

**Fig. 3.**
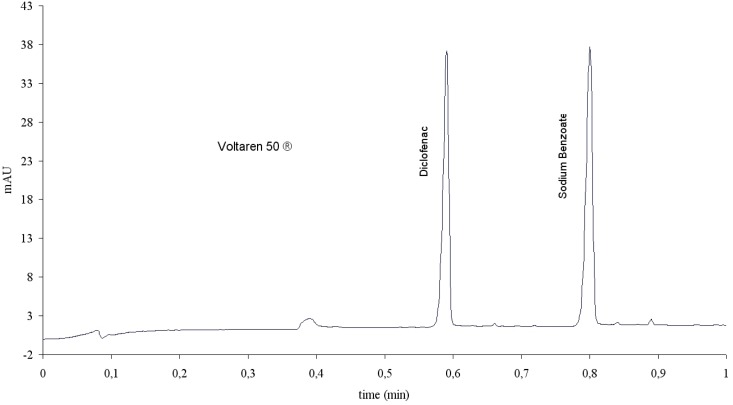
Electropherogram of a Voltaren 50 mg® sample, obtained with method 2.

**Tab. 1.** Quantitative results from all samples by method 2.

**Formulation**	**Weight (g)**	**Content declared (mg)**	**Amount found (mg)**	**% RSD two times n = 5**	**Amount found % mean**
Voltaren Dispers 50 I	0.257	50	49.4	0.7%	98.8%
Voltaren Dispers 50 II	0.255	50	48.8	0.6%	97.6%
Voltaren 50 I	0.215	50	51.1	0.9%	102.1%
Voltaren 50 II	0.213	50	50.2	0.6%	100.4%
Voltaren 50 III	0.213	50	51.6	0.8%	103.2%
Voltaren ret.	0.299	100	101.0	2.1%	101.0%
Voltaren Emulgel	1.7741	9.9688 mg/g calculated as sodium salt	16.7	2.6%	92%
Voltaren Amp.	2.763	75 mg / 3ml	67.2	1%	97.3%
Control 1		35.9	35.5	0.8%	98.8%
Control 2		53.1	53.8	1.1%	101.3%
